# N-Phenylquinazolin-2-amine Yhhu4952 as a novel promotor for oligodendrocyte differentiation and myelination

**DOI:** 10.1038/s41598-018-32326-0

**Published:** 2018-09-19

**Authors:** Xueli Yu, Gang Cheng, Lei Zhang, Yu Zhang, Qing Wang, Mengxue Zhao, Limin Zeng, Youhong Hu, Linyin Feng

**Affiliations:** 1 CAS Key Laboratory of Receptor Research and Department of Neuropharmacology,Shanghai Institute of Materia Medica, 555 Zu Chongzhi Road, Shanghai, 201203 China; 2 University of Chinese Academy of Sciences, No.19A Yuquan Road, Beijing, 100049 China; 3 College of Pharmaceutical Sciences, Zhejiang University, Hangzhou, 310058 China

## Abstract

Oligodendrocytes are a type of glial cells that ensheath multiple neuronal axons and form myelin. Under pathological conditions, such as multiple sclerosis (MS), inflammatory damage to myelin and oligodendrocytes leads to demyelination. Although the demyelinated regions can partially resolve functional deficits through remyelination, however, as the disease progresses, remyelination typically becomes incomplete and ultimately fails. One possible explanation for this failure is the activation of the Notch pathway in MS lesions, which impedes oligodendrocyte precursor cells (OPCs) at maturation. This leads to a potential target for remyelination. Here, we have identified a compound Yhhu4952 that promoted the maturation of cultured OPCs in a dose-dependent and time-dependent manner. Neonatal rats showed a significant increase in the expression of myelin basic protein (MBP) and the prevalence of mature oligodendrocytes in the corpus callosum after Yhhu4952 treatment. The compound was also effective in promoting remyelination in cuprizone-induced demyelination model and improving severity scores in experimental autoimmune encephalomyelitis (EAE) model. Mechanism studies revealed that Yhhu4952 promotes OPC differentiation through the inhibition of the Jagged1-Notch1 pathway. These findings suggest Yhhu4952 is potentially useful for proceeding oligodendrocyte differentiation and remyelination.

## Introduction

OPCs originate from the germinal zones of the cortex and the spinal cord. These cells can also divide and migrate throughout the entire central nervous system^1,2^. Their typical characteristics are platelet-derived growth factor receptor alpha (PDGFRα) and the chondroitin sulfate proteoglycan NG2^3–5^. Although OPCs can produce mature oligodendrocytes throughout their entire lifespan^6^, the regeneration of myelin typically fails under pathological conditions. This is likely due to the decreased OPC recruitment and differentiation^7^.

Multiple sclerosis (MS) is a demyelinating disease associated with the activation of the immune system, loss of myelin, and impairment of the axonal integrity^8–10^. Since inflammatory processes are the main cause for myelin destruction, the majority of MS therapies are immunomodulatory drugs aimed at reducing the relapse rate^11^. Unfortunately, these drugs have a very limited effect on remyelination or axonal repair. Although cell transplantation therapy has been considered for enhancing the remyelination^12–14^, purification and generation of the transplanted cells, adverse events such as cell dosing, administration route, immunological rejection, etc., need further investigation^15,16^. Therefore, facilitating myelin repair from endogenous OPCs is considered a promising strategy for MS drug development.

Since the myelin repair process is largely dependent on OPCs^17,18^, remyelination failure may be related to the shortage^19^ or inadequate recruitment of OPCs^20^. However, a major challenge for MS is the failure of OPCs to differentiate into mature myelin-forming oligodendrocytes, although OPCs are abundant in MS lesions^21^. This defect is likely due to the accumulation of various inhibitory signals for myelination^22,23^. For example, the polysialylated neural cell adhesion molecule (PSA-NCAM)^24^ and the LINGO-1^25,26^ have been identified as inhibitors for oligodendrocyte-axon interaction. Additionally, Notch1 receptor is a known negative regulator of OPC differentiation. Notch1 and its downstream effector Hes5 are localized on OPCs, while the Notch ligand Jagged1 is expressed on axons and reactive astrocytes^27^. The activation of the Jagged1-Notch1 pathway has been implicated in the inhibition of OPC differentiation in MS lesions^28^. Thus, targeting these regulating signals is of great importance in developing remyelination therapies.

Significant progress has been made in facilitating endogenous remyelination by high-throughput drug screening. For example, benztropine, clemastine^29^, miconazole and clobetasol^30^ were identified as facilitators of OPC differentiation. Despite this “new used old drugs” strategy, discovering novel scaffolds as lead compounds may provide new perspectives in remyelination drug development.

## Results

### Yhhu4952 promotes OPC differentiation

**Figure 1:**
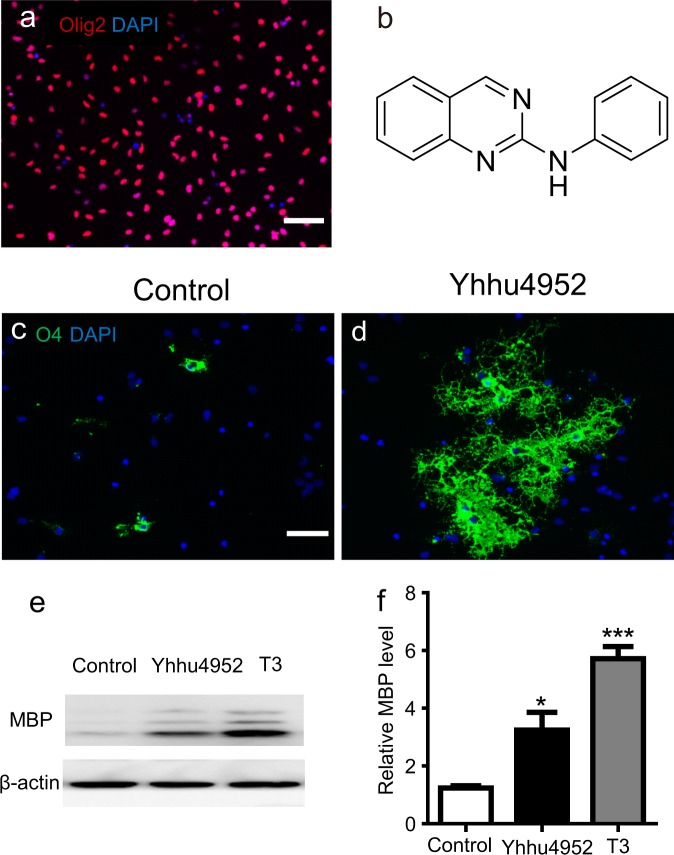
Yhhu4952 promotes OPC differentiation. (**a**) OPCs staining with Olig2 (red), the nuclei were labelled with DAPI (blue). (**b**) The chemical structure of Yhhu4952. (**c,d**) OPCs were treated with Yhhu4952 for 3 days then staining with O4 (green). (**e,f**) Western blot assay of MBP expression after 6 days culture. Data are analyzed using one-way ANOVA and displayed as mean ± SEM of three independent experiment. *p < 0.05, ***p < 0.001 compared with Control. Scale bar = 50 μm. Full-length blots are presented in Supplementary Fig. 4a.

To identify compounds promoting OPC differentiation, the research group of Professor Youhong Hu designed and synthesized a series of compounds aimed at promoting OPC maturation. To investigate the effects of these compounds on oligodendrocyte lineage, we established primary cultures of rat OPCs, and the typical characteristics of cultured OPCs were verified by immunostaining for the OPC marker Olig2, showing nearly 90% of the total cells as Olig2 positive (Fig. 1a). Then, we performed compound screening based on the morphometric assay of oligodendrocyte late progenitor marker O4. The results showed that Yhhu4952 (Fig. 1b), which was previously described as a weak cannabinoid receptor type 2 (CB2) agonist^31^, is a facilitator that promotes OPC maturation. Upon the withdrawal of mitogens (PDGF-AA and bFGF) to trigger differentiation, 5 μM Yhhu4952 were added in the culture for 3 days. The expression of O4 was evaluated by immunostaining, and Yhhu4952-treated OPCs evidently increased cell complexity and exhibited more branches compared with control, suggesting that Yhhu4952 stimulated OPC differentiation (Fig. 1c,d). Triiodothyronine (T3), a known enhancer of OPC maturation and myelination, was used as positive control for evaluating OPC differentiation. To further confirm the facilitative effect of Yhhu4952, 5 μM Yhhu4952 and 0.3 μM T3 were added to the OPC culture for 6 days, and the mature oligodendrocyte marker MBP protein expression was examined by western blotting. The results showed that Yhhu4952 significantly increased MBP expression by nearly two-fold (Fig. 1e,f, also Supplementary Fig. 4a). Collectively, these data indicated that Yhhu4952 could promote OPC differentiation.

### Yhhu4952 inhibits the proliferation of OPCs *in vitro*

**Figure 2:**
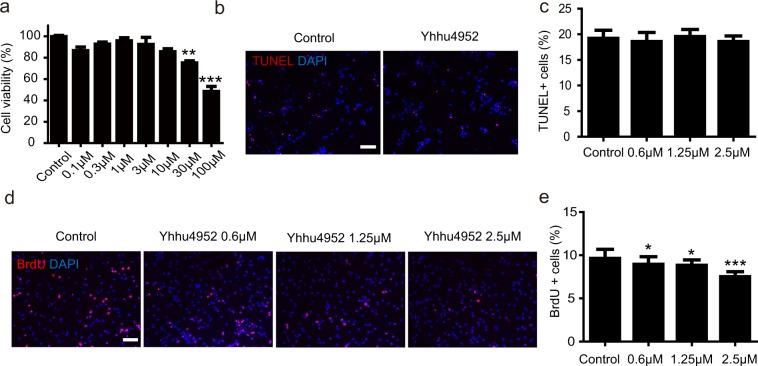
Yhhu4952 inhibits the proliferation of OPCs without affecting cell viability or apoptosis. (**a**) Effects of Yhhu4952 on OPCs viability were assessed by Cell Counting Kit-8. (**b**) TUNEL staining (red) following various Yhhu4952 concentration treatment. (**c**) Quantification of TUNEL^+^/DAPI cells. (**d**) BrdU incorporation assay was performed in OPC culture. Cells were immunostained with BrdU (red). (**e**) Quantification of BrdU^+^/DAPI cells. Data are analyzed using one-way ANOVA and displayed as mean ± SEM of three independent experiment. *p < 0.05, **p < 0.01, ***p < 0.001 compared with control. Scale bar = 50 μm.

To examine the cytotoxicity of Yhhu4952, a cell counting kit-8 assay (Fig. 2a) was performed by adding various concentrations of Yhhu4952 (0.1, 0.3, 1, 3, 10, 30 and 100 μM) to OPC culture. The results indicated that Yhhu4952 did not significantly affect the cell viability under 10 μM but this compound was cytotoxic above 30 μM. Next, we used a TUNEL assay to further investigate whether the compound could induce OPCs apoptosis, and the results revealed that Yhhu4952 did not affect OPCs apoptosis within 2.5 μM (Fig. 2b,c). Furthermore, a BrdU incorporation assay was performed to evaluate the potential effect of Yhhu4952 on the proliferation of OPCs. Concentrations ≧ 0.6 μM Yhhu4952 caused a significant reduction of BrdU-positive cells in a dose-dependent manner (Fig. 2d,e). Taken together, these data suggested Yhhu4952 decreased the proliferative capability of OPCs *in vitro*.

### Yhhu4952 induces differentiation and maturation of oligodendrocytes *in vitro*

**Figure 3:**
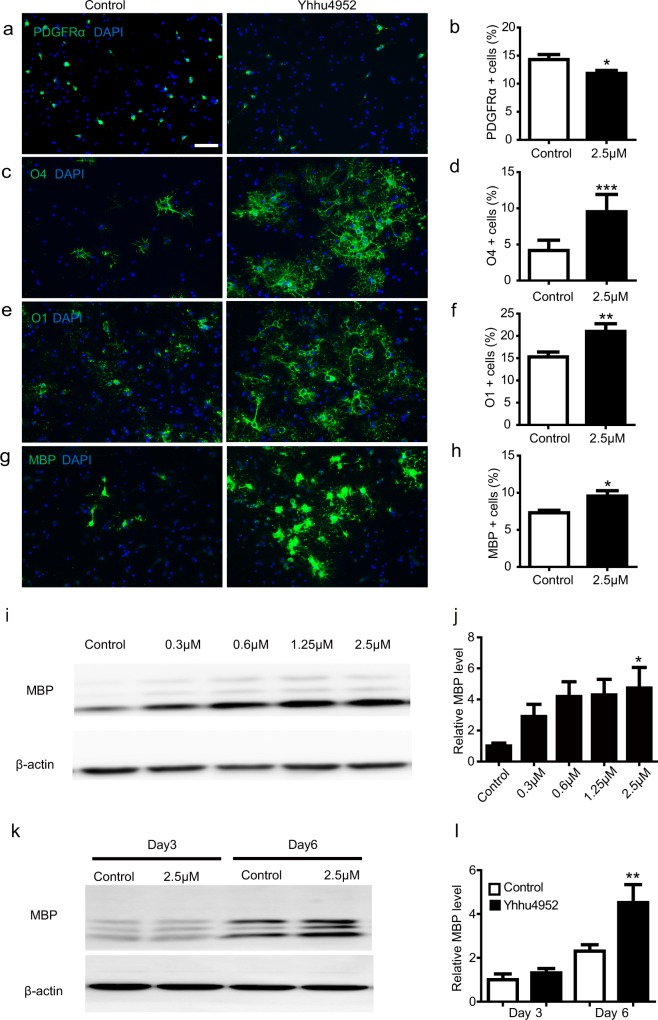
Yhhu4952 promotes OPC differentiation into mature oligodendrocytes in a dose-dependent and time-dependent manner. (**a**–**h**) OPCs were treated with 2.5 μM Yhhu4952 for 6 days. OPC differentiation were assessed by staining with PDGFRα (green), O4 (green), O1 (green), MBP (green). The immunopositive cells/DAPI were quantified. (**i,j**) OPCs were treated with various concentration of Yhhu4952 for 6 days. (**k,l**) OPCs were treated with 2.5 μM Yhhu4952 for 3 or 6 days, the MBP protein expression levels were examined by western blot. Data are represented as mean ± SEM and analyzed using Student’s t test or one-way ANOVA. (Three independent experiments were performed). *p < 0.05, **p < 0.01, ***p < 0.001 compared with relative control. Scale bar = 50 μm. Full-length blots are presented in Supplementary Fig. 4b,c.

The maturation of oligodendrocytes is a highly regulated process characterized by four stages: oligodendrocyte early progenitors, oligodendrocyte late progenitors, immature oligodendrocytes, and mature oligodendrocytes^32^. Many regulators temporally and spatially participate in this process and coordinate the timing of maturation^33^. Previously, we observed the facilitative effect of Yhhu4952 on OPCs (Fig. 1), however, it was unclear at which stage and to what extent Yhhu4952 contributes to OPC maturation. To evaluate the effect of Yhhu4952 on oligodendrocyte development, OPCs were treated with Yhhu4952 at 2.5 μM for 6 days followed by immunostaining for these four stage-specific markers, and the percentage of the immunopositive cells was calculated. As OPCs are a bipotent glial cell type in the CNS that can differentiate into both astrocytes and oligodendrocytes, we also quantified the percentage of GFAP labelled astrocytes, there were about 14% GFAP-positive cells in control and 12.3% after Yhhu4952 treatment (Supplementary Fig. 1). The number of PDGFRα labelled early progenitors significantly decreased after Yhhu4952 treatment (Fig. 3a,b), while the number of O4-positive cells (Fig. 3c,d), O1-positive cells (Fig. 3e,f), and MBP-positive cells (Fig. 3g,h) significantly increased, indicating that Yhhu4952 could promote the differentiation of oligodendrocyte early progenitors differentiate into mature oligodendrocytes. Additionally, to explore the influence of various concentrations of Yhhu4952 and different treatment time on OPCs, 0.3 ~ 2.5 μM Yhhu4952 were added in OPC culture or treated with 2.5 μM Yhhu4952 for 3 and 6 days. MBP expression was assayed by western blotting. The results revealed that the levels of MBP were significantly elevated at 2.5 μM in a dose-dependent manner (Fig. 3i,j, Supplementary Fig. 4b) and increased about two-fold compared to relative control at day6 (Fig. 3k,l, Supplementary Fig. 4c). The above results revealed that Yhhu4952 could promote OPC differentiation in a dose- and time-dependent manner.

### Yhhu4952 accelerates myelination *in vivo*

**Figure 4:**
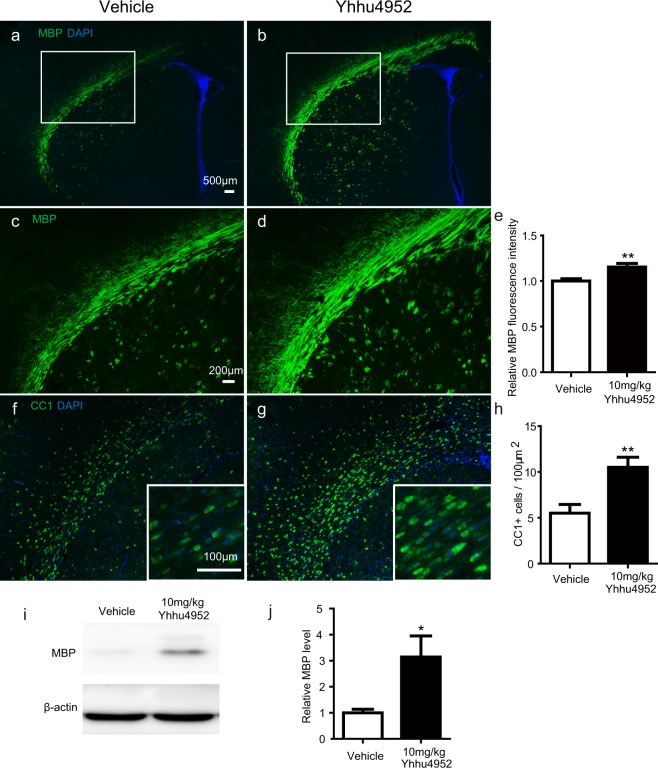
Yhhu4952 promotes myelination in the developmental model. (**a,b**) Representative immunofluorescence staining with MBP (green) and DAPI (blue) of lateral corpus callosum (CC) of the neonatal P10 rats with vehicle or Yhhu4952 treatment. (**c**–**e**) Quantification of total MBP (green) fluorescence intensity in CC lateral area. (**f**–**h**) Quantification of CC1^+^ cells per 100 μm^2^ in CC area. (**i,j**) Quantification of MBP protein expression levels in CC area. Data are represented as mean ± SEM (n = 4 per group) and analyzed using Student’s t test. *p < 0.05, **p < 0.01 versus vehicle. Full-length blots are presented in Supplementary Fig. 4d.

Based on the previous findings that Yhhu4952 promoted OPC maturation *in vitro*, the involvement of Yhhu4952 in the timing of animal development has not been verified by *in vivo* studies. We first investigated whether Yhhu4952 crosses blood-brain barrier with penetration into brain parenchyma, an i.p. dose of 10 mg/kg Yhhu4952 were given at different time points (0, 10 min, 30 min, 1 h, 3 h and 5 h), rats (n = 3 for each time point) were euthanized, the plasma and brain samples were immediately collected for further analyses. The results showed that Yhhu4952 rapidly enters the CNS and reaches a maximal concentration of 4.05 μg/g in the brain, which is approximately 2.53 times the maximal plasma concentration (Supplementary Fig. 2). To address whether Yhhu4952 facilitates oligodendroglial development, postnatal day 2 rats were intraperitoneally injected with 10 mg/kg Yhhu4952 or 1% Tween80-normal saline as a vehicle for 8 days. We detected immunofluorescence intensity of MBP (showed in rectangle area, Fig. 4a,b) and quantified CC1 labelled mature oligodendrocyte numbers in lateral corpus callosum. The analysis revealed that at a dosage of 10 mg/kg, Yhhu4952 significantly upregulated the MBP intensity approximately 1.2 times versus vehicle (Fig. 4c-e) and increased CC1-positive oligodendrocyte numbers (Fig. 4f-h). Furthermore, quantification of the western blot data demonstrated that Yhhu4952 treatment significantly upregulated MBP expression in the corpus callosum by approximately 3 times versus vehicle (Fig. 4i,j, Supplementary Fig. 4d). These findings indicated Yhhu4952 could induce precocious myelination *in vivo* by promoting OPC maturation.

### Efficacy of Yhhu4952 in the cuprizone model and the EAE model

**Figure 5:**
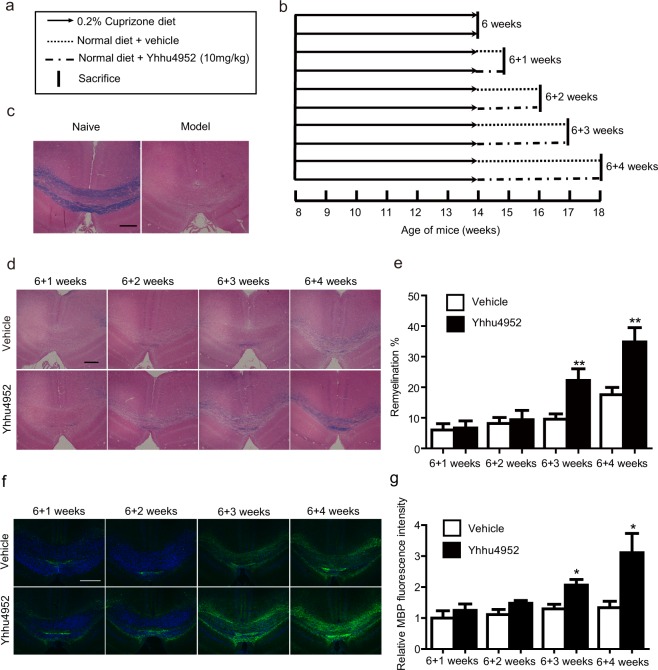
Efficacy of Yhhu4952 in the cuprizone model. (**a,b**) Schematic representation of animal treatment. The C57BL/6 mice (n = 4 per group) were fed with 0.2% (w/w) of cuprizone containing diet for 6 weeks to induce demyelination. Following cuprizone withdraw, mice were treated with 10 mg/kg Yhhu4952 or normal saline for another 4 weeks. (**c**) Representative images (n = 6 images each from 4 mice per group) of corpus callosum region stained with Luxol fast blue and eosin after six weeks of cuprizone diet. (**d,e**) Quantifications of remyelinating areas. (**f,g**) Quantification of the relative MBP (green) fluorescence intensity in corpus callosum. Data are represented as mean ± SEM and analyzed using Student’s t test. *p < 0.05, **p < 0.01. Scale bar = 400 μm.

To determine whether Yhhu4952 could promote myelination under pathological conditions, we first investigated the myelinating ability of Yhhu4952 using cuprizone model, which is a T-cell-independent toxic model. Cuprizone exposure induces the apoptosis of mature oligodendrocytes and disrupts myelin formation, thus leading to demyelination. In the present study, 0.2% (w/w) cuprizone was integrated into the normal diet of eight-week old C57BL/6 mice and maintained for six weeks to induce demyelination. Subsequently, the normal diet was reintroduced. The mice were intraperitoneally dosed with either Yhhu4952 (10 mg/kg) or 1% Tween80-normal saline as vehicle daily for another 4 weeks, and subsets were euthanized weekly (Fig. 5a,b). After staining with Luxol fast blue (LFB) and eosin, the remyelination region was then quantified. Demyelination was evidently observed in the corpus callosum after 6 weeks of the cuprizone diet (Fig. 5c). In the following weeks, spontaneous remyelination can be observed. During week 7 and 8, both vehicle and Yhhu4952 treatment group showed limited remyelination. However, when compared with the vehicle group, significant remyelination was observed by the end of the week 9 and 10, as the proportion of the myelinated area increased from 9.58 ± 0.90% to 22.21 ± 3.89% and from 17.55 ± 4.07% to 34.82 ± 2.20%, respectively (Fig. 5d,e). Furthermore, we evaluated and quantified myelin expression in the corpus callosum by immunostaining with MBP, the results demonstrated that consistent with the previous LFB study, Yhhu4952 significantly promoted MBP expression after 3 weeks of Yhhu4952 administration (Fig. 5f,g). These findings indicated that Yhhu4952 might directly act on and facilitate the maturation of OPCs because this model is largely mediated by toxicity rather than provoking immune responses.

**Figure 6:**
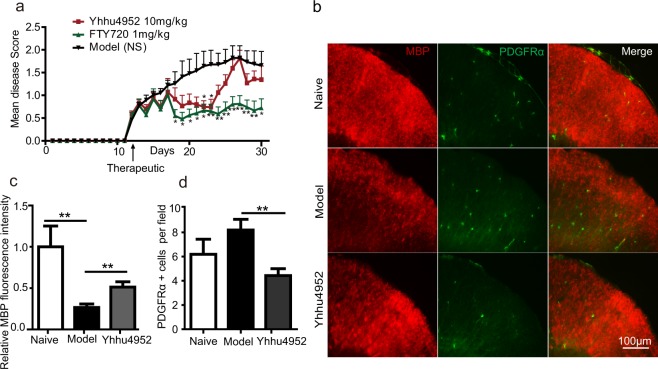
Efficacy of Yhhu4952 in the EAE mouse model. (**a**) EAE was induced in C57BL/6 mice (n = 8 per group), disease scores were evaluated daily. (**b**) Immunofluorescence staining of MBP and PDGFRα in the spinal cords of EAE mice (n = 10 images each from 3 mice per group) on post-immunization day 22. (**c,d**) Quantification of the relative MBP fluorescence intensity and PDGFRα-positive cells per field in white matter. Data are represented as mean ± SEM and analyzed using Student’s t test. *p < 0.05, **p < 0.01 compared with model.

EAE is a well-described animal model of MS and is commonly used to determine the effectiveness of immunosuppressive or pro-myelinating agents^34,35^. FTY720 is an oral immunomodulatory drug for the treatment of MS and is often used as a reference compound for studying EAE^36^. We further assessed the activity of Yhhu4952 using a MOG35 – 55-induced EAE mouse model. 10 mg/kg Yhhu4952, 1 mg/kg FTY720 or 1% Tween80-normal saline (NS) were administered at the onset of EAE signs (day12), and the results revealed that the model mice exhibited typical EAE disease course with scores steadily increasing while FTY720 effectively inhibited the progression of EAE during observation period, which was never >1.0. Treatment with Yhhu4952 improved disease scores from day12 and significantly reduced EAE severity at day 22 and 23. However, it were unable to continue suppressing EAE at later periods as relapses were clearly observed from day 24 (Fig. 6a). To verify the beneficial effect of Yhhu4952 on remyelination, we stained the spinal cord of EAE mice with MBP and PDGFRα on post-immunization day 22, and the relative MBP fluorescence intensity and number of PDGFRα-labelled OPCs per field were calculated (Fig. 6b). A substantial decrease of MBP protein expression was found in the EAE model mice, while Yhhu4952 partially resolved demyelination by increasing MBP expression (Fig. 6c). Increased OPC numbers in the demyelinated area could be observed in the model mice, indicating that OPCs were recruited to the lesion site but failed to further differentiate. Compared with the model group, PDGFRα-positive cell numbers were significantly decreased after Yhhu4952 treatment (Fig. 6d), suggesting Yhhu4952 promotes remyelination by facilitating OPC maturation at the lesion site. These observations indicate that Yhhu4952 accelerates remyelination at the lesion site and improves EAE severity, but fails to prevent EAE mice from repeated immune attacks.

### Yhhu4952 promotes OPC differentiation through inhibition of Jagged1-Notch1 pathway

**Figure 7:**
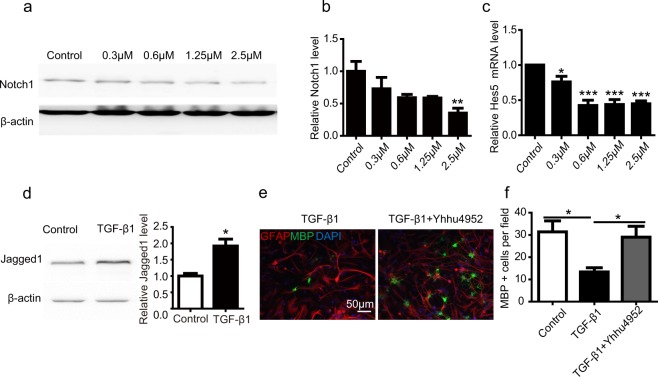
Yhhu4952 promotes the differentiation of OPCs through the inhibition of Jagged1-Notch1 pathway. (**a,b**) Quantification of Notch1 protein expression after various concentration of Yhhu4952 treatment. (**c**) Real-time PCR demonstrating the Hes5 mRNA levels after various concentration of Yhhu4952 treatment. (**d**) Quantification of TGF-β1-induced Jagged1 expression levels. (**e**) Astrocyte-oligodendrocyte cocultures were stained with MBP (green) and GFAP (red). (**f**) Quantification of MBP^+^ cells per field. Data are expressed as mean ± SEM (three independent experiment were performed), and statistically analyzed using one-way ANOVA. *p < 0.05, **p < 0.01, ***p < 0.001 compared with relative control. Full-length blots are presented in Supplementary Fig. 4e,f.

Notch signalling is a highly conserved pathway that is considered fundamental within organism development, cell proliferation and cancer biology^37^. The inhibition of Notch1 by siRNA potentiated OPC maturation and restricted the cell proliferation^38^. Consistent with the present study, our previous data implicated Yhhu4952 might involve in Notch1-mediated OPC differentiation. To validate this hypothesis, we evaluated the protein expression of the Notch1 receptor, as well as the mRNA levels of its downstream effector Hes5 in cultured OPCs. Western blot results showed that Yhhu4952 suppressed Notch1 protein expression in a dose-dependent manner (Fig. 7a,b, Supplementary Fig. 4e), and Hes5 mRNA expression was significantly reduced at the concentrations ≧ 0.3 μM (Fig. 7c). These data demonstrated that Yhhu4952 inhibited the Notch1 signalling pathway in OPCs.

The suppressive role of the Jagged1-Notch1 pathway on remyelination has been suggested by evidence that Notch1-positive oligodendrocytes are exclusively expressed in demyelination plaques, and reactive astrocytes are the major sources of Jagged1 in MS lesions^39^. The contact-mediated activation of Jagged1-Notch1 pathway induces Hes5 expression in OPCs, which inhibits the maturation of oligodendrocytes^27,40–42^. Moreover, the cytokine TGF-β1 upregulated in MS and can specifically induce Jagged1 expression in human astrocytes^40,43^. Based on this, we generated an astrocytes and OPCs coculture system to confirm whether Yhhu4952 is involved in the inhibition of the Jagged1-Notch1 signalling pathway. We first quantified Jagged-1 protein expression on astrocytes culture after 5 days of Yhhu4952 treatment, and no significant difference was found between Control and Yhhu4952 (Supplementary Fig. 3). OPCs were plated onto astrocytes, treated with 10 ng/ml of TGF-β1 for 24 h, and the induction of Jagged1 by TGF-β1 in rat astrocytes was detected by western blotting. (Fig. 7d, Supplementary Fig. 3f). Next, 2.5 μM of Yhhu4952 was added to the culture for another 6 days after TGF-β1 wash out. The cocultures were fixed and immunostained with GFAP and MBP, and the percentage of MBP-positive cells was subsequently calculated. As expected, the TGF-β1-treated group displayed an inhibitory effect on OPC differentiation as the number of MBP-positive cells were significantly decreased versus those of the untreated control, while the Yhhu4952 treatment group showed the reversed effects of TGF-β1 induced differentiation suppression (Fig. 7e,f). The above results suggest that Yhhu4952 promotes the maturation of OPCs by inhibiting the Jagged1-Notch1 pathway.

## Discussion

Remyelination can occur when the tissue is exposed to demyelination insult^44^. The recruitment and the differentiation of OPCs are key steps in myelin repair. Despite the declined efficiency of remyelination with the age^45,46^, the inadequate differentiation of OPCs is due to the inhibitory environment^47^. Therefore, discovering compounds that facilitate endogenous repair has great potential for the treatment of demyelinating diseases. After screening a library of synthesized compounds, Yhhu4952, a compound reported as a low-potency partial CB2 receptor agonist^31^, was identified as a novel facilitator of OPC maturation (Fig. 1). We have shown that Yhhu4952 inhibits the proliferation and promotes the differentiation of OPCs *in vitro*. Moreover, Yhhu4952-treated rat pups revealed a precocious myelination in early postnatal rats, and Yhhu4952 enhanced remyelination in the cuprizone model and modulated the course of EAE. Mechanistically, we demonstrated that Yhhu4952 promoted OPC differentiation through the inhibition of the Jagged1-Notch1 signalling pathway.

The facilitating properties of Yhhu4952 in OPC cultures prompted us to evaluate its therapeutic relevance in animal models of MS. We showed that Yhhu4952 treatment accelerated remyelination in the cuprizone model. Since cuprizone-induced demyelination is largely mediated by toxicity rather than immune attacks, therefore this finding suggests Yhhu4952 might directly act on oligodendrocytes. EAE is another common animal model that shares many pathological similarities to MS, and most therapies or modifying agents were extensively evaluated on the basis of EAE studies^48^. In the present study, Yhhu4952 evidently attenuated disease severity and promoted remyelination at an earlier stage, but relapses were clearly observed from day 24 and Yhhu4952 failed to continue suppressing disease progress at a later period. Interestingly, CB2 receptors are largely expressed on immune cells^49^ and mediate immunomodulatory and anti-inflammatory actions^50^. CB2 receptors are up-regulated in the activated microglia cells of EAE mice^51^, and the administration of Gp1a,a CB2 agonist, significantly reduced EAE mice severity^52^. Therefore, as a weak CB2 receptor agonist, Yhhu4952 might also exhibit a mild beneficial effect on EAE mice, however, this compound was insufficient to suppress disease progression. Myelin repair is a complicated process that involved in the proliferation, migration and differentiation of OPCs into myelin-forming oligodendrocytes^53^. Based on previous findings, it is likely that the reduced EAE severity is due to both the facilitated effect of Yhhu4952 on OPCs and mild immunomodulatory effects at the lesion site. One of the reasons for remyelination failure is repeated episodes of demyelination insults leading to the exhaustion of “OPCs pools” with too few cells available for recruitment^54^, which might also explain the inevitable relapse in the EAE model.

Although we cannot rule out the possibility that other unrecognized targets may exist, we provide compelling evidence that Yhhu4952-induced OPC differentiation is mediated, at least in part, through the inhibition of the Notch1 pathway. Notch signalling has been extensively studied over the past decades, and Notch1 has facilitated a variety of developmental processes by controlling cell proliferation without affecting apoptosis^55^. The inhibition of Notch1 receptor and its effector Hes5 promoted OPC differentiation at the expense of OPCs proliferation^41,56^. The present findings showed that Yhhu4952 increased MBP expression and decreased the percentage of BrdU-positive cells in a dose-dependent manner, consistent with previous studies (Figs 2, 3). *In vitro* studies confirmed this hypothesis, as the expression of Notch1 receptor and its downstream transcription factor Hes5 was significantly decreased (Fig. 7a–c). The Notch ligand Jagged1 has been found in reactive astrocytes and axons in MS lesions and can be specifically induced by TGF-β1^40^, and activation of Jagged1-Notch1 pathway impeded the remyelination in MS lesions^27,40–42^. To simulate the activation of the Jagged1-Notch1 pathway, we performed astrocyte-oligodendrocyte coculture as previously described^38^. As expected, TGF-β1-treated culture exhibited restricted OPC differentiation, whereas Yhhu4952 significantly reversed this inhibitory effect while increasing the MBP labelled mature oligodendrocyte prevalence (Fig. 7e,f). This result further confirmed the involvement of Notch1 in modulating OPC maturation.

It has been reported that the Notch1 receptor null heterozygotes mutant mice exhibited transient premature myelination and elevated myelin genes expression during the first two weeks of postnatal life^56^. In line with this, we evaluated the effect of Yhhu4952 on neonatal rat myelination development, and found that Yhhu4952-treated rat pups contained greater numbers of CC1 labelled mature oligodendrocytes and a significant increase of MBP expression in the corpus callosum (Fig. 4). Previous studies have identified benztropine as a facilitator for myelin regeneration that also inhibits the Notch1 expression. The administration of benztropine was also effective in increasing remyelination in cuprizone model and improving EAE severity score^57^. Additionally, blocking Notch pathway by γ-secretase in experimental autoimmune encephalomyelitis (EAE) model showed the reduction of axon damage and enhanced remyelination^58^. For instance, semagacestat and Quercetinare are both Notch signalling inhibitors that significantly enhance remyelination and improve disease score in EAE model^59^, therefore Notch signalling may be a potential target for MS treatment. However, none of these compounds have been evaluated in MS patients or yielded satisfying results in clinical trials^60^.

Cannabinoid CB1 and CB2 receptors are distributed throughout the body and involved in a series of physiological processes. CB1 receptors, as central cannabinoid receptors, are found at a remarkably high density in brain regions associated with pain processing, emotion, and motor behaviour. CB2 receptors are mainly expressed by all haematopoietic and microglial cells in the central nervous system (CNS)^49^. The administration of CB2 receptor agonists JWH133 not only attenuated inflammatory pain in rat models^61^ but also modulated the microglia release of pro-inflammatory cytokines in brain^62^. More importantly, CB2 agonists also displayed beneficial effects in experimental models of EAE and Alzheimer disease^52,63^. Hence targeting CB2 receptors has emerged as a potential therapeutic approach to treat inflammatory and autoimmune diseases.

To our knowledge, the present study is the first to demonstrate that Yhhu4952, a weak CB2 receptor agonist, promoted OPC maturation both *in vitro* and *in vivo* by suppression of the Notch signalling pathways. However, more intensive studies are needed to determine whether compounds with similar structures are capable of inhibiting Notch signalling and whether this facilitative effect on OPCs is shared by CB2 agonists. In summary, Yhhu4952 has great potential for providing new perspectives in the development of MS drugs and serves as a potential lead candidate for the treatment of demyelinating diseases.

## Methods

### Compounds

The compound Yhhu4952 has been reported as a novel agonist for the cannabinoid CB2 receptor^31^, and was prepared from commercial available 2, 4-dichloroquinazoline via selective reduction protection and aniline substitution by Dr. Youhong Hu research group. Yhhu4952 was dissolved in DMSO for *in vitro* studies or 1% Tween80 in normal saline for *in vivo* assay.

### Animals

All the animals were purchased from Shanghai Laboratory Animal Center (Shanghai, China). Animals were housed in environmentally controlled conditions (22 °C, 12 h dark/12 h light cycle and a relative humidity of 60%) with facilitate access to standard laboratory chow and water. All of the experimental procedures were carried out in accordance with the Care and Use of Laboratory Animals (National Institutes of Health) and protocols were approved by the Animal Research Ethics Board of Shanghai Institute of Materia Medica, Chinese Academy of Sciences.

### Oligodendrocyte progenitor cell culture

OPCs were generated using previously described methods^64–67^. In brief, neural stem cells (NSCs) were isolated from the hippocampus region of embryonic day 14 rat and cultured in DMEM/F12 medium with 2% of B27, 1% N2 supplements and 20 ng/ml EGF and bFGF (Invitrogen Corp. USA) for 7 days. To generate OPCs, neurospheres were dissociated using StemPro Accutase (Invitrogen Corp. USA) and plated on uncoated culture dishes in oligodendrocyte culture medium (2% B27-supplemented DMEM/F12 medium) with 10 ng/ml bFGF and PDGF-AA growth factor (Invitrogen Corp. USA). Small adherent oligospheres were formed. After 2 passages, highly enriched OPCs can be maintained. For maturation experiment, OPCs were plated on 10 μg/ml poly-D-lysine coated coverslips at the density of 2 × 10^5^ cells/ml, differentiation can be triggered by withdrawing bFGF and PDGF-AA growth factors.

### Immunofluorescence staining

Cultured cells or brain slices (30 μm) were fixed with 4% paraformaldehyde followed by three washes in PBS and permeabilized with 0.1% Triton X-100 for 10 min (except for O4, O1 immunostainning) then blocked in 10% goat serum (Invitrogen Corp. USA) for 1 h following incubation with the primary antibody overnight at 4 °C. Primary antibodies were diluted in blocking solutions as follows: mouse anti-MBP (Biolegend, USA; 1:500), mouse anti-APC, CC1 clone (Millipore, USA; 1:500), mouse anti-O4 (R&D Systems Inc, USA, 1:800), mouse anti-O1 (R&D Systems Inc, USA, 1:800), rabbit anti-PDGFRα (Cell Signalling Technology, USA; 1:500), rabbit anti-glial fibrillary acidic protein (GFAP, Biolegend, USA; 1:500) mouse anti-BrdU (Sigma St Louis, USA; 1:500). The tissue or cells were then washed with PBS and incubate in Alexa Flour-conjugated secondary antibodies (Invitrogen Corp. USA; 1:500) for 1 h. Images were obtained using Olympus photomicroscope and analyzed using Image-Pro plus 6.0 software.

### Western blot

Cells were harvest in lysis buffer on ice. Sample proteins were loaded on SDS-PAGE gel and blotted onto PVDF membranes. After blocking in 5% (w/v) non-fat milk for 1 h, proteins were probed with primary antibodies at 4 °C overnight. Primary antibodies were diluted in 5% bovine serum albumin blocking buffer as follows: rabbit anti-Jagged1 (Cell Signalling Technology, USA; 1:500), rabbit anti-Notch1 (Cell Signalling Technology, USA; 1:1000), mouse anti-MBP (Biolegend, USA; 1:1000). Membranes were then incubated in horseradish peroxidase-conjugated secondary antibody for 1 h, the immunoreactive proteins can be detected by chemiluminescence ECL agents (Millipore, USA).

### Cell viability assay

The quantification of viable cells were determined by Cell Counting Kit-8 (Dojindo Molecular Technologies, Japan). OPC suspension (100 µL/well) was added to a 96-well plates and incubated overnight at 37 °C in a humidified incubator containing 5% CO_2_. Cultures were then treated with various concentrations of Yhhu4952 for 24 h, 10 μl of cell counting kit-8 solution was added to each well and incubated for another 4 h. The absorbance at 450 nm was measured by NOVO star MicroPlate Reader (BMG Technologies, Germany).

### TUNEL assay

Terminal deoxynucleotidyl transferase mediated d-UTP nick end labelling (TUNEL) assay was performed using *in situ* cell death detection kit (Roche Group, Switzerland) following the manufacturer’s protocols. OPCs were treated with various concentrations of Yhhu4952 for 24 h then fixed with 4% paraformaldehyde following incubation in TUNEL reaction solution mixture at room temperature for 1 h. The cell nuclei were stained with DAPI and analyzed using Image-Pro plus 6.0 software.

### BrdU incorporation assay

Dissociated OPCs were plated on poly-D-lysine coated coverslips and treated with Yhhu4952 in oligodendrocyte culture medium for 48 h before incubated with 10 μM BrdU (Sigma St Louis, USA) for 12 h. Cultures were fixed with 4% paraformaldehyde and incubated with 2 M HCl for 30 min then neutralized with borate buffer (0.1 M, PH 8.5) for another 30 min, immunostaining with BrdU was performed following immunocytochemistry method.

### Developmental myelination

Postnatal day 2 Sprague-Dawley rats were administered 10 mg/kg Yhhu4952 or vehicle (1% Tween80 in normal saline) by intraperitoneal injections for 8 days. Rats were anesthetized with 5% chloral hydrate and immediately collected for western blot analysis (4 rats per group) or transcardially perfused with 4% paraformaldehyde (4 rats per group). After last injection, rat brains were dehydrated in 30% sucrose for 4 days then embedded in OCT. Serial coronal sections (30 μm) were obtained using cryostat microtome (Leica, Wetzlar, Germany). The sections were proceed to immunostaining. The relative MBP expression intensity and the CC1-positive cells per 100 μm^2^ in the corpus callosum regions were quantified using Image-Pro plus 6.0 software.

### The cuprizone model for demyelination

Eight-week-old female C57BL/6 mice were fed with 0.2% w/w cuprizone (Sigma St Louis, USA) thoroughly mixed in powdered normal rodent chew for 6 weeks to induce demyelination, then put on normal chew for another 4 weeks. Yhhu4952 was dissolved in normal saline at final concentration of 10 mg/kg, the vehicle group were given 1% Tween80 in normal saline. Mice were injected intraperitoneally with Yhhu4952 or vehicle every day and sacrificed at different time points (0, 1, 2, 3 and 4 weeks). Mice brains were extracted, paraffin-embedded and sectioned (5 μm) using Ultra-Thin Semiautomatic Microtome (Leica, Wetzlar, Germany) then stained with eosin and Luxol fast blue (Sigma St Louis, USA) for histopathological analysis. Images were obtained by Leica Microsystems, and the percentage of remyelination area were quantified using Image-Pro plus 6.0 software.

### Induction of experimental autoimmune encephalomyelitis (EAE)

To induce EAE, eight-week-old female C57BL/6 mice (8 rats per group) were subcutaneously immunized with 200 μg MOG_35 – 55_ in incomplete Freund’s adjuvant (Sigma, St. Louis. USA) containing 5 mg/ml of heat-killed Mycobacterium tuberculosis (strain H37RA; Difco, USA) and intraperitoneally injected 200 ng pertussis toxin (Enzo Life Sciences. USA) in 100 μl PBS at day 0 and day 2. 10 mg/kg Yhhu4952, 1 mg/kg FTY720 or 1% Tween80-normal saline (NS) were intraperitoneally injected since the onset of immunization. Clinical EAE severity was graded by another two independent observers following the standard 0 – 5 EAE grading scale: 0, natural; 1, tail limpness or waddling gait; 2, hind limb weakness; 3, paralysis of one limb; 4, paralysis of two limbs; 5, death. Mice (3 rats per group) were anesthetized with 5% chloral hydrate and transcardially perfused with 4% paraformaldehyde on post-immunization day 22. Spinal cords of mice were dehydrated in 30% sucrose, and sectioned (20 μm) for MBP and PDGFRα immunostaining. The relative MBP fluorescence intensity and the number of PDGFRα labelled OPCs per field were quantified using Image-Pro plus 6.0 software.

### Real-time polymerase chain reaction

Cells were lysed using TRIzol reagent (Invitrogen Corp. USA), total ribonucleic acid (RNA) were isolated and reverse-transcribed. Expression of mRNA levels were determined by SYBR Premix EX Taq kit (Takara Bio Inc, Japan) following the manufacturer’s instructions. The primers were used as follows: β-actin, forward primer: 5′-GGAGATTACTGCCCTGGCTCCTA-3′ and reversed primer: 5′-GACTCATCGTACTCCTGCTTGCTG-3′; Hes5, forward primer: 5′-ACCTGAAGCACAGCAAAGC-3′ and reversed primer 5′-GCCGCTGGAAGTGGTAAA-3′; Transcripts were normalized to endogenous control β-actin and quantitated by comparative C_t_ method.

### Astrocyte-oligodendrocyte coculture

The coculture of astrocytes and oligodendrocytes were performed following the reported methods^38^. Rat OPCs were plated onto astrocyte-enriched cultures and treated with 10 ng/ml TGF-β1 (Peprotech, USA) for 24 h, following TGF-β1 withdraw, 2.5 μM Yhhu4952 were added in culture for another 5 days. Cultured cells were fixed with 4% paraformaldehyde and immunostained with GFAP and MBP. The number of MBP-positive cells per field were quantified using Image-Pro plus 6.0 software.

### Statistical analysis

The student’s t test was used to compare two experimental groups. One-way ANOVA was used for multiple comparisons. p < 0.05 was considered statistically significant.

## Electronic supplementary material

Supplementary Information

## Data Availability

The datasets generated during and/or analysed during the current study are available in the figshare repository https://figshare.com/.
