# Supplementary material for: N-Phenylquinazolin-2-amine Yhhu4952 as a novel promotor for oligodendrocyte differentiation and myelination
Source: Sci Rep. 2018 Sep 19;8:14040. doi: 10.1038/s41598-018-32326-0 (PMC6145871; doi:10.1038/s41598-018-32326-0)
Supplement: Supplementary file 1 — Supplementary Information [file 41598_2018_32326_MOESM1_ESM.docx]

N-Phenylquinazolin-2-amine Yhhu4952 as a novel promotor for oligodendrocyte differentiation and myelination.

Xueli Yu^1,2^, Gang Cheng^3^, Lei Zhang^1,2^, Yu Zhang^1,2^, Qing Wang^1,2^, Mengxue Zhao^1,2^, Limin Zeng^1,2^, Youhong Hu^3*^ and Linyin Feng^1,2*^

^1^CAS Key Laboratory of Receptor Research and Department of Neuropharmacology,

Shanghai Institute of Materia Medica, 555 Zu Chongzhi Road, shanghai, 201203, China

^2^University of Chinese Academy of Sciences, No.19A Yuquan Road, Beijing 100049, China

^3^College of Pharmaceutical Sciences, Zhejiang University, Hangzhou, 310058, China

^*^Corresponding Author: Linyin Feng, Youhong Hu

The Address and E-mail of Corresponding Author: 555, Zu Chong Zhi Road, Shanghai, People’s Republic of China, 201203. [lyfeng@simm.ac.cn](mailto:lyfeng@simm.ac.cn); [yhhu@simm.ac.cn](mailto:yhhu@simm.ac.cn)


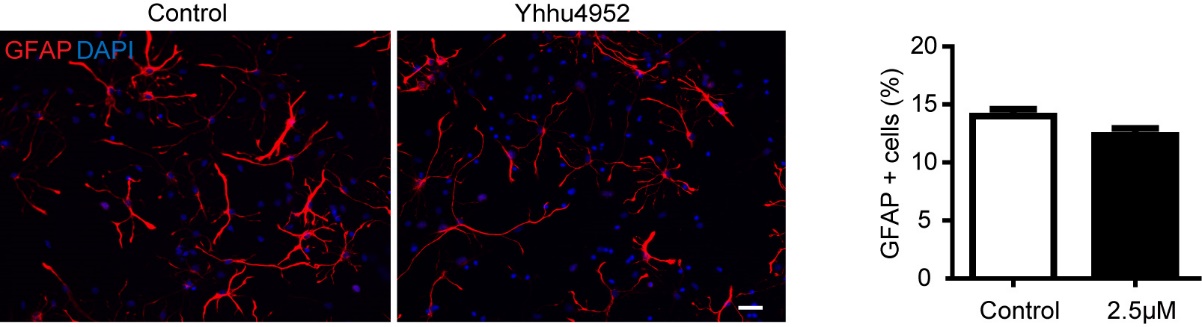


Supplementary Figure 1. Quantification of GFAP^+^/DAPI cells after Yhhu4952 treatment on OPCs for 6 days. Data are represented as mean ± SEM (three independent experiment were performed), and analyzed using Student’s t test. Scale bar=50μm.


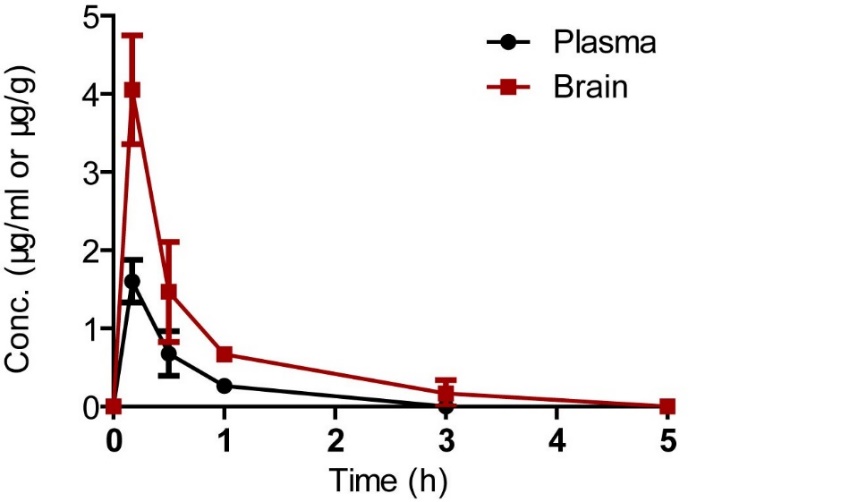


Supplementary Figure 2. The plasma (black), brain (red) concentration-time profiles of Yhhu4952. Following an i.p. dose of 10 mg/kg at different time points (0, 10min, 30min, 1h, 3h, 5h), rats (n=3 for each time point) were euthanized, the plasma and brain samples were immediately prepared for Yhhu4952 concentration determination.


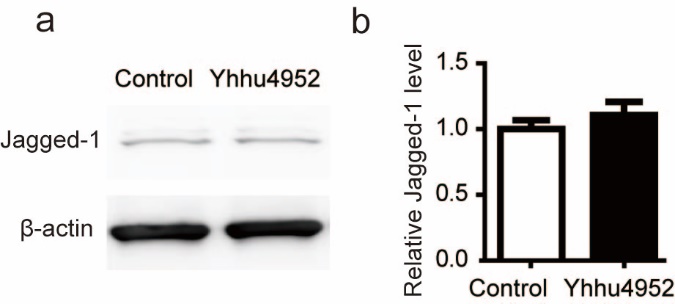


Supplementary Figure 3. Quantification of Jagged-1 protein expression levels on astrocytes after Yhhu4952 treatment. Data are represented as mean ± SEM (three independent experiment were performed), and analyzed using Student’s t test.


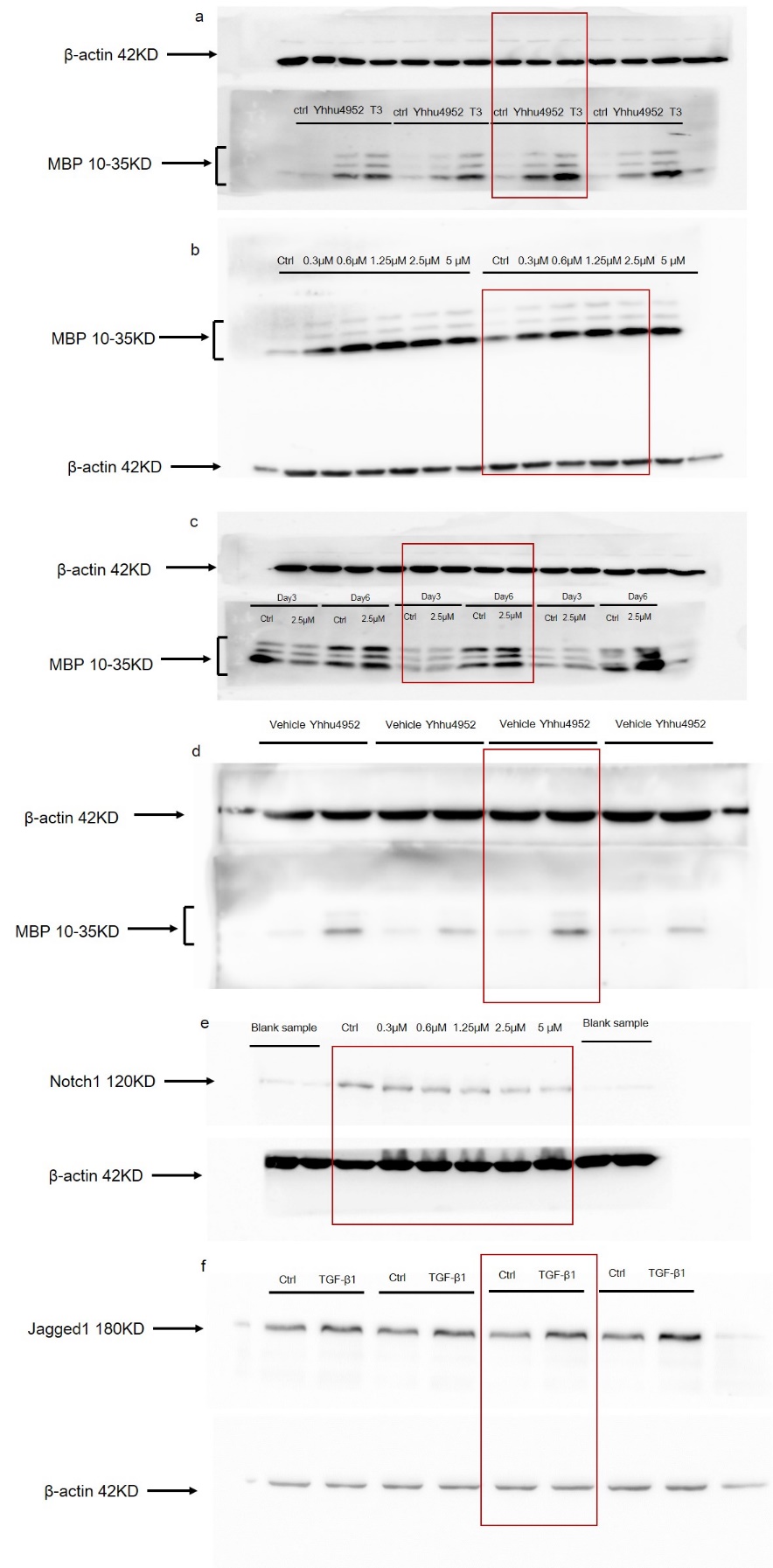


Supplementary Figure 4. Source full-length Western blots used in cropped panel in figures 1, 3, 4 and 7. (a) Blots corresponding to Figure 1. (b,c) Blots corresponding to Figure 3. (d) Blots corresponding to Figure 4. (e,f) Blots corresponding to Figure 7. Slight contrast and brightness optimizations were applied in (e) in order to improve visibility of the cropped parts.
